# Interferon-*γ* and Interleukin-10 Responses during Clinical Malaria Episodes in Infants Aged 0–2 Years Prenatally Exposed to* Plasmodium falciparum*: Tanzanian Birth Cohort

**DOI:** 10.1155/2018/6847498

**Published:** 2018-07-30

**Authors:** Boniphace Sylvester, Dinah B. Gasarasi, Said Aboud, Donath Tarimo, Siriel Masawe, Rose Mpembeni, Göte Swedberg

**Affiliations:** ^1^Department of Parasitology and Medical Entomology, School of Public Health and Social Sciences, Muhimbili University of Health and Allied Sciences, P.O. Box 65001, Dar es Salaam, Tanzania; ^2^Department of Microbiology and Immunology, School of Medicine, Muhimbili University of Health and Allied Sciences, P.O. Box 65001, Dar es Salaam, Tanzania; ^3^Department of Obstetrics and Gynaecology, School of Medicine, Muhimbili University of Health and Allied Sciences, P.O. Box 65001, Dar es Salaam, Tanzania; ^4^Department of Epidemiology and Biostatistics, School of Public Health and Social Sciences, Muhimbili University of Health and Allied Sciences, Dar es Salaam, Tanzania; ^5^Department of Medical Biochemistry and Microbiology, Biomedical Centre, Uppsala University, P.O. Box 582, Uppsala, Sweden

## Abstract

**Background:**

Infants born to mothers with placental malaria are prenatally exposed to* Plasmodium falciparum *antigens. However, the effect of that exposure to subsequent immune responses has not been fully elucidated. This study aimed at determining the effect of prenatal exposure to* P. falciparum *on Interleukin-10 and Interferon-*γ* responses during clinical malaria episodes in the first 24 months of life.

**Methods:**

This prospective cohort study involved 215 infants aged 0-2 years born to mothers with or without placental malaria. Enzyme-linked immunosorbent assay (ELISA) was used to determine levels of IL-10 and IFN-*γ* in infants and detect IgM in cord blood. Data were analyzed using SPSS version 20.

**Findings:**

Geometric mean for IFN-*γ* in exposed infants was 557.9 pg/ml (95% CI: 511.6-604.1) and in unexposed infants it was 634.4 pg/ml (95% CI: 618.2-668.5) (P=0.02). Mean IL-10 was 22.4 pg/ml (95% CI: 19.4-28.4) and 15.1 pg/ml (95%CI: 12.4-17.6), respectively (P=0.01).

**Conclusions:**

Prenatal exposure to* P. falciparum *antigens significantly affects IL-10 and IFN-*γ* responses during clinical malaria episodes in the first two years of life.

## 1. Background

*Plasmodium falciparum *malaria is a disease of public health importance and causes mortality of at least one million children below five years of age annually [1–4]. Infants who are born to mothers with placental malaria are in utero exposed to malaria infected erythrocytes or their soluble products with subsequent fetal immune sensitization or tolerance [5, 6]. The* in utero *effects on the immune cells due to early exposure to the parasite antigen may persist in childhood, leading to subsequent hyporesponsiveness or immunosuppression to the same parasite antigen and hence increased susceptibility to malaria infection [7]. This suggests that* in utero *exposure to* P. falciparum *may modulate the development of fetal protective immune cells culminating into disruption in the balancing of T helper 1 (Th1) and T helper 2 (Th2) immune responses as reported elsewhere [8]. Interferon-*γ* (IFN-*γ*) is a Th1 proinflammatory cytokine that has been demonstrated to have antiparasitic effect [9, 10], while IL-10 is a Th2 cytokine that has been demonstrated to be an important immunoregulatory anti-inflammatory cytokine [11]. Although a number of studies have been carried out to explain the phenomenon of prenatal exposure to the parasite antigen and its subsequent effect on the development of protective immune responses, the findings remain inconclusive. This study was designed to assess the effect of prenatal exposure to* P. falciparum *antigens on selected cytokines (IL-10 and IFN-*γ*) response during clinical malaria episodes in infants aged zero to two years. The two cytokines IFN-*γ* and IL-10 were selected since IFN-*γ* is a key Th1-derived inflammatory cytokine that is important for clearance of intracellular parasites like* P. falciparum*, while its biological activity can be inhibited by Th2-derived IL-10 that inhibits natural killer cells and macrophage activity and has been demonstrated to be an important anti-inflammatory cytokine [12]. Other factors that could confound the effect of prenatal exposure to* P. falciparum *on cytokine responses such as season of birth, infant birth weight, use of Insecticide Treated bed Nets (ITNs), gravidity, parasite density, and timing of malaria episodes were also assessed.

## 2. Methods

### 2.1. Study Area

Detailed description of the study area appears elsewhere [13]. Briefly, the study was conducted in Rufiji district on the Southeast Coast of Tanzania. This district, in terms of malaria endemicity, was earlier characterized as a holoendemic area. This level of endemicity may now have changed following the rigorous National Malaria Control Program campaigns with distribution of Insecticide Treated Nets (ITNs). The current malaria prevalence in this district is 4.8% [14].

### 2.2. Study Design, Sample Population, and Recruitment of Research Participants

Detailed description of the study design and participants recruitment process was described in an earlier report [13]. Briefly, this was a prospective birth cohort study and the research participants were recruited after delivery. A total of 215 mother-infant pairs were recruited. 50 infants born to mothers with placental malaria were categorized as exposed to* P. falciparum*, while 165 infants born to mothers without placental malaria were categorized as unexposed. The infants were followed at three monthly intervals (regular scheduled visits) and whenever they fell sick up (irregular unscheduled visits) to the age of two years. During the follow-up, all clinical malaria episodes were recorded and respective blood samples were collected for determination of IL-10 and IFN-*γ*.

### 2.3. Establishment of Placental Malaria and Collection of Cord Blood and Infant Peripheral Blood during Follow-Ups

Detailed procedures for collection of placental tissues, storage of placental tissues, histopathology, and follow-ups were reported elsewhere [13]. Briefly, after delivery, placental tissue was collected and preserved in 10% neutral-buffered formalin. The fixed tissues were then subsequently processed and examined using histopathological techniques to establish placental malaria status. Cord blood (3mls) samples were collected after delivery in order to establish the baseline levels of IL-10 and IFN-*γ*.

During follow-ups, recruited infants were examined for clinical signs at the health facilities and a clinical malaria episode was described as fever (≥37.5°C) plus parasitaemia established through microscopic examination of 10% Giemsa stained smears. All clinical malaria episodes encountered by recruited infants born to mothers with placental malaria and without placental malaria were recorded, respective blood samples were collected, and the infants were managed at the health facilities. Following clotting of collected cord and infant peripheral blood samples, sera were separated in aliquots of 200 *μ*l and 100 *μ*l each, respectively. Aliquot sera were initially stored at −20°c at the field site and later transported in a cool box packed with frozen ice to −80°c freezers at Muhimbili University of Health and Allied Sciences (MUHAS) Immunology Laboratory for determination of IL-10 and IFN-*γ*.

### 2.4. Determination of Baseline IL-10 and IFN-*γ* Levels in Cord Blood and in Infant Peripheral Blood during Clinical Malaria Episodes and Assessment of In Utero Sensitization to PfMSPI-19 and PfMSPII

IFN-*γ* and IL-10 levels were quantified in cord blood sera as baseline and in peripheral blood sera collected during clinical malaria episodes using ELISA in separate plates as per manufacturer's instructions (Abcam®, UK). All materials and prepared reagents were equilibrated at room temperature prior to use in the assay. 100 *μ*l of IL-10 and IFN-*γ* standards and test sera were added to appropriate wells and were covered well and incubated for two and a half hours at room temperature. The solutions were discarded and the plate was washed four times with washing buffer. The complete removal of liquid at each step was essential to ensure good performance of the assay. After the last wash, any remaining buffer solution was removed by aspiration and the plate was inverted and blotted against a paper towel. 100 *μ*L of working biotinylated IL-10 and IFN-*γ* detection antibodies was added to respective wells and incubated for one hour at room temperature while shaking gently. This was followed by discarding the solution and washing three times. 100 *μ*l of working horseradish peroxidase streptavidin solution was added to each well and was incubated for 45 minutes at room temperature while shaking gently. The solution was then discarded and washed three times. 100 *μ*L of Tetramethylbenzidine (TMB) one-step substrate reagent was added to each well and incubated for 30 minutes at room temperature. Finally, 50 *μ*l of stop solution was added to each well and optical density (OD) was read at 450 nm. The serial diluted concentrations of standard and their corresponding OD were used to plot a standard curve with standard concentrations on* x*-axis and mean absorbance on* y*-axis. The best fit straight line was drawn and the concentrations of IL-10 and IFN-*γ* in test samples were obtained and recorded in pg/ml. Determinations of IgM specific to PfMSP1-19 and PfMSPII antigens were done in cord blood sera samples. Presence of IgM in cord blood sera specific for PfMSP1-19 and PfMSPII antigens defined in utero sensitization while their absence defined in utero nonsensitization. The detailed ELISA procedures have been reported elsewhere [15]. The positivity of the test sample for IgM was defined at a cutoff point (mean + 3SD) of a negative control group born and living in Sweden.

### 2.5. Data Analysis

Data were analyzed using IBM SPSS version 20. Analysis was done on log transformed data in order to use parametric tests in the analysis. Comparisons of mean levels of IL-10 and IFN-*γ* during clinical malaria episodes between exposed and unexposed infants were carried out using independent-samples* t*-test. ANOVA was used to assess the mean difference in levels of IL-10 and IFN-*γ* in exposed sensitized, exposed nonsensitized, and unexposed infants. Multiple linear regression model was used to assess the effect of prenatal exposure to* P. falciparum *and other factors (gravidity, birth weight, and season of birth) on levels of IL-10 and IFN-*γ*. Significance of difference was judged at 95% confidence level and a P value < 0.05.

## 3. Results

### 3.1. Social Demographic Characteristics of the Study Population

Detailed description of the social demographic characteristics of the study population was reported elsewhere [13]. Briefly, gender, season of birth, and use of Insecticide Treated bed Nets were not significantly different (P>0.05) between exposed and unexposed infants; however, exposed infants had significantly lower birth weight compared to the unexposed infants (P<0.05).

### 3.2. Levels of IL-10 and IFN-*γ* during Clinical Malaria Episodes and In Utero Sensitization to Pf MSPI-19 and PfMSPII

Recruited infants were thoroughly examined, and infants who were found to have clinical malaria episodes did not have other diseases at the time of blood sample collection for determination of IL-10 and IFN-*γ*.* P. falciparum *was found to be the only* Plasmodium *species diagnosed using light microscopic technique. The details of timing and number of clinical malaria episodes were reported elsewhere [12]. Briefly, the mean number of clinical malaria episodes during the first 2 years of life, among infants born to pm+ mothers, was 0.51 episodes/infant compared to those born to pm− mothers with 0.30 episodes/infant. The geometric means for baseline IL-10 in cord blood for exposed and unexposed infants were 5.4 pg/ml (95% CI: 4.5-6.4) and 5.7 pg/ml (95% CI: 4.7-6.7), respectively, and the difference was not statistically significant (P=0.74). The geometric means for baseline IFN-*γ* in cord blood for exposed and unexposed were 407.1 pg/ml (95% CI: 366.5-447.6) and 390.2 pg/ml (95% CI: 355.5-424.9), respectively, and the difference was not statistically significant (P=0.54, Table 1). Geometric means of IL-10 in exposed and unexposed infants during clinical malaria episodes were 22.4 pg/ml (95% CI: 19.4-28.4) and 15.1 pg/ml (95% CI: 12.4-17.6), respectively, and the difference was statistically significant (P=0.01). The geometric means for IFN-*γ* during clinical malaria episodes in exposed and unexposed infants were 557.9 pg/ml (95% CI: 511.6-604.1) and 634.4 pg/ml (95% CI: 618.2-668.5), respectively, and the difference was statistically significant (P=0.02). Out of 50 infants who were born to mothers with placental malaria, 5 (10%) had specific IgM to PfMSP1-19 and PfMSP2 and were categorized as sensitized to* P. falciparum *antigens and the rest (90%) were nonsensitized. All infants born to mothers without placental malaria were not prenatally exposed to* P. falciparum *and had no detectable IgM against PfMSP1-19 and PfMSPII in cord blood sera. The ANOVA for levels of IL-10 and IFN-*γ* in the three subgroups of infants, exposed sensitized, exposed nonsensitized, and unexposed infants, indicated that the cytokine response for the three groups was different and this difference was statistically significant (P<0.05, Table 2).

### 3.3. Effect of Prenatal Exposure to* P. falciparum* and Other Factors on IL-10 and IFN-*γ* Responses during Clinical Malaria Episodes

All clinical malaria episodes were recorded, respective cytokines levels were measured, and geometric means were compared in exposed and unexposed groups of infants in the first two years of life.

Timing to clinical malaria episodes has been adjusted for in a multivariate analysis. Prenatal exposure to malaria was significantly associated with IL-10 (P<0.05) and IFN-*γ* responses (P<0.05) during clinical malaria episodes, while other factors did not significantly associate with IL-10 and IFN-*γ* responses (Table 3).

## 4. Discussion

Immunopathology of malaria disease involves proinflammatory cytokines such as tumor necrosis factor-alpha (TNF-alpha), Interleukin-12 (IL-12), and IFN-*γ* which may mitigate parasite development and stimulate monocyte phagocytosis towards clearance of parasitized red blood cells [16, 17]. IL-10 is known to be anti-inflammatory, such that it antagonizes the effects of IFN-*γ* and it is a major mediator for the production of acute phase reactants in the pathogenesis of malaria [16]. This implies that the balance between proinflammatory cytokines and anti-inflammatory cytokines is likely to play a crucial role in the resolution of malaria infection and may determine whether the host response becomes protective or nonprotective [18].

Prenatal exposure to* P. falciparum *antigens has been demonstrated to affect the development of fetal immune cells [1] and affects the susceptibility of infants to malaria infection [5, 13, 19–22]. A number of* in vitro *studies have demonstrated that cord blood lymphocytes from malaria exposed placenta are nonresponsive when stimulated with similar parasite antigens. This nonresponsiveness has been ascribed to early exposure to the parasite antigens and is implicated to play a negative role in subsequent development of protective immune responses against the same parasite antigen. This phenomenon of prenatal sensitization has been demonstrated in filarial and other helminthes infections [23–26].

On the basis of these findings, this study was designed to further characterize the effect of prenatal exposure to* P. falciparum *antigens on cytokine response during clinical malaria episodes focusing on IL-10 and IFN-*γ*, which are anti-inflammatory and pro-inflammatory cytokines, respectively [16, 27, 28]. The results of this prospective birth cohort study have shown that prenatal exposure to* P. falciparum *antigens is significantly associated with low IFN-*γ* and high IL-10 responses during clinical malaria episodes in the first two years of life.

The low IFN-*γ* response during clinical malaria episodes in exposed infants in this study may partly explain the effect of prenatal exposure to* P. falciparum *on antimalarial immunity in infants prenatally exposed to* P. falciparum *antigens, since IFN-*γ* has been demonstrated to effectively induce parasite killing by monocytes and neutrophils [29]. This observation implies that low production of IFN-*γ* during clinical malaria episodes may negatively affect the infant's ability to clear the malaria parasites through the cytokine responses and indicates a possible disruption of the biological roles of IFN-*γ*, including but not limited to activation of macrophages for phagocytosis and major histocompatibility complex class II (MHCII) molecule expression, which are important in antimalarial immunity [30].

The observations in this study may be supported by previous finding that indicated increased susceptibility of infants born to mothers with placental malaria when naturally challenged with malaria infection. These infants succumbed to clinical malaria infection at a shorter time from birth and experienced more attacks of clinical malaria episodes in their first two years of life, which may suggest compromised protective immunity development in these infants [13]. Furthermore, the downregulated IFN-*γ* responses during clinical malaria episodes observed in this study corroborate previous findings that demonstrated that prenatal exposure to* P. falciparum *downregulated Th1 responses* in utero *and that possibly later in life could contribute to susceptibility of infants to malaria infection in early childhood [31].

Early fetal exposure to parasite antigens modulates the development of fetal T cells leading to fetal immunosensitization or immunological tolerance which is indicated by hyporesponsiveness. The effect of immunological tolerance following in utero exposure to* P. falciparum *tends to suppress fetal expression of Th1 and CD8+ T cells as demonstrated by a study conducted in Gabon [32]. The similar pattern of cytokine response (low IFN-*γ* and high IL-10) in Gabon's in vitro study with cord blood cells and the present in vivo study in infants' peripheral blood plasma suggests that the effect of fetal T cell exposure to the parasite antigens persists to the first two years of life.

In a multivariate analysis, after adjusting for other factors (gravidity, season of birth, parasite density, timing of malaria episodes, and birth weight), which may influence acquisition of malaria infection and subsequent host immune response, the results showed that prenatal exposure was the only factor that was significantly associated with IFN-*γ* and IL-10 responses. The geometric mean level of IFN-*γ* during clinical malaria episodes in the first two years of life in exposed infants was significantly lower than that in unexposed infants. The low IFN-*γ* responses in exposed infants indicate the effect of prenatal exposure to* P. falciparum *on antimalarial immunity in infants prenatally exposed to* P. falciparum *antigens. IFN-*γ* has been demonstrated to effectively induce parasite killing by monocytes and neutrophils [29]; therefore, this observation implies that low production of IFN-*γ* during clinical malaria episodes may negatively affect the infant's ability to clear the malaria parasites, a finding that may be corroborated by an earlier study [33] that demonstrated the presence of hyperparasitaemia in exposed infants during clinical malaria episodes.

The high levels of IL-10 during clinical malaria episodes accompanied with downregulated IFN-*γ* production observed in this study are likely to create a conducive host environment for the survival of* P. falciparum*. In terms of malaria pathogenesis, it may be hypothesized that* P. falciparum *parasites evade parasite clearance by modulating the host immune system such that IL-10 that has been associated with susceptibility to malaria [27] becomes elevated and IFN-*γ* that has antiparasitic effect becomes downregulated, thus creating a host environment that may favor propagation of* P. falciparum. *The high IL-10 response during clinical malaria episodes demonstrated in infants born to mothers with placental malaria in this study may also indicate a possible inhibition of Th2 cytokine response accompanied with high production of IL-10, leading to disruption of Th1/Th2 balance, curtailing the inhibition of* P. falciparum *replication, a role played by IFN-*γ* [34].

The results of this study therefore shade more light on the effects of prenatal exposure to the parasite antigen on host susceptibility and development of protective immunity against* P. falciparum *malaria. In this study, the majority of infants who were born to mothers with placental malaria were nonsensitized to* P. falciparum *antigens. This observation may possibly explain the downregulation of IFN-*γ* during clinical malaria episodes, which potentially lowers antiparasitic effects and makes the infants more vulnerable to malaria infection. Furthermore, this study shows that cytokine responses are an important component of immunity against* P. falciparum *infection in childhood. Therefore, strategies towards design of malaria vaccine candidates may among other aspects consider a vaccine candidate that will restore the optimal Th1/Th2 balance that will be able to potentiate the host antiparasitic effects, restrain the propagation of parasites, and mitigate the occurrence of clinical malaria episodes.

## 5. Conclusions

Prenatal exposure to* P. falciparum *significantly affects IFN-*γ* and IL-10 cytokine response during clinical malaria episodes in infants aged zero to two years.

## Figures and Tables

**Table 1:** Geometric mean of IL-10 and IFN-*γ* response at baseline (in cord blood) and during clinical malaria episodes.

Cytokine	Mean pg/ml (95% CI) of infants born to pm+ mothers (n=50)	Mean pg/ml (95% CI) of infants born to pm- mothers (n=165)	P value
BL IL-10	5.4 (4.5-6.4)	5.7 (4.7-6.7)	0.74
BL IFN-*γ*	407.1 (366.5-447.6)	390.2 (355.5-424.9)	0.54
DCME 1L-10	22.4 (19.4-28.4)	15.1 (12.4-17.6)	0.001
DCME IFN-*γ*	557.9 (511.6-604.1)	634.4 (618.2-668.5)	0.002

BL: baseline levels, DCME: during clinical malaria episodes, CI: confidence interval. The statistical P value was obtained using independent-samples *t*-test.

**Table 2:** Geometric mean of IL-10 and IFN-*γ* in exposed sensitized, exposed nonsensitized, and unexposed infants during clinical malaria episodes: Analysis of Variance (ANOVA).

Cytokine	ESI Mean (95% CI) pg/ml	ENSI Mean (95% CI) pg/ml	NEI Mean (95% CI) pg/ml	P value
IL-10	25.4 (5.2-51.3)	21.8 (15.2-28.4)	15.1 (12.4-17.6)	0.029
IFN-*γ*	576.1 (368.5-623.8)	540.2 (499.1-581.4)	634.4 (600.2-668.5)	0.008

CI: confidence interval, ESI: exposed sensitized infants, ENSI: exposed nonsensitized infants, NEI: nonexposed infants. Statistical P value was obtained using ANOVA.

**Table 3:** Effect of prenatal exposure to *P. falciparum*, gravidity, season of birth, parasite density, timing of malaria episodes, and birth weight on IL-10 and IFN-*γ* responses during clinical malaria episodes: Multiple linear regression model (MLRM).

**Factor**	**Univariate analysis**		**Multivariate analysis**	
**IL-10**	Coefficient (95% CI)	P value	Coefficient (95% CI)	P value

IUE *P. falciparum*	0.17 (0.050-0.288)	0.006	9.176 (3.11-15.20)	0.004
Gravidity	0.04 (-0.086-0.157)	0.558	5.722 (-0.023-11.468)	0.063
Season of birth	-0.04 (0.201-0.118)	0.604	0.401 (-6.575-7.378)	0.909
Infant birth weight	-0.035 (-0.185-0.116)	0.649	2.315 (-4.422-9.052)	0.496
Parasite density	3.41 (0.616-6.209)	0.015	-4.689 (-16.687-7.31)	0.438
Timing to malaria episodes	-1.205 (-7.088-4.675)	0.684	-1.846 (-4.382-2.581)	0.739

**IFN-**γ				

IUET *P. falciparum*	-0.056 (-0.096- -0.016)	0.007	-90.92 (151.697- -30.145	0.003
Gravidity	-0.006 (-0.046-0.035)	0.771	-38.832 (-96.415-18.750)	0.183
Season of birth	-0.042 (-0.094-0.010)	0.115	-74.873 (-144.79-4.956)	0.076
Infant birth weight	0.023 (-0.026-0.073)	0.035	13.822 (-53.69-81.34)	0.684
Parasite density	0.06 (-0.015-0.027)	0.587	77.522 (-42.726-197.77)	0.202
Timing to malaria episodes	-0.008 (-0.020-0.004)	0.197	-12.651 (-23.32-7.762)	0.235

IUE *P. falciparum*:in utero exposure to *P. falciparum*, SD: standard deviation, CI: confidence interval.
